# Artificial Intelligence Capability and Organizational Creativity: The Role of Knowledge Sharing and Organizational Cohesion

**DOI:** 10.3389/fpsyg.2022.845277

**Published:** 2022-03-09

**Authors:** Na Li, Yapeng Yan, Yuting Yang, Anwei Gu

**Affiliations:** ^1^School of Management, Changchun University, Changchun, China; ^2^School of Business, Dalian University of Technology, Dalian, China; ^3^School of Management, Jilin University, Changchun, China

**Keywords:** artificial intelligence, organizational creativity, knowledge sharing, cohesion, capability

## Abstract

The rapid development of artificial intelligence (AI) has brought many opportunities and challenges to organization. Some studies have shown that AI can improve organizational creativity. However, the existing research lacks an effective transformation path. This paper makes an innovative approach from the perspective of knowledge sharing, establishes an integration model of artificial intelligence capability, knowledge sharing and organizational creativity. Based on 189 questionnaire data, we use multi-level regression analysis and bootstrap method to analyze the influence mechanism. The results show that artificial intelligence has a positive effect on knowledge sharing, knowledge sharing has a positive effect on organizational creativity, knowledge sharing mediates the relationship between artificial intelligence and organizational creativity, and organizational cohesion has a positive moderating effect on the relationship between artificial intelligence and knowledge sharing. The results supplement the existing research on the relationship between artificial intelligence capability and organizational creativity, expand the theoretical boundary and application space from the perspective of knowledge sharing at the organizational level, and provide reference for organizations to improve creativity.

## Introduction

Artificial intelligence (AI) has been regarded by many as the next source of business value, as a strategic technology leading a new round of technological revolution and industrial transformation. Its rapid development brings many opportunities and challenges to organizations. To acquire key artificial intelligence technologies (Bughin et al., 2017). Artificial intelligence technology can enhance entrepreneurs’ judgment and decision-making ability, is the development trend of The Times, is a powerful tool to promote social development and progress. As a result, artificial intelligence technology is now becoming increasingly important (Townsend et al., 2018).

Knowledge sharing (KS) is very important for firms to achieve strategic goals because it can help firms generate new sources of knowledge through collaboration and creation, significantly update firms’ problem-solving skills and increase awareness of sharers’ decision-making process. Creativity is the basic feature of human intelligence and also the inevitable challenge of artificial intelligence. At present, scholars have applied artificial intelligence into practice and made wonderful movie trailers that generally require the assistance of human creativity, promoting the possibility of artificial intelligence to increase personal expertise and creativity (Smith et al., 2017).

In order to solve the impact of artificial intelligence on organizational creativity, some scholars creatively proposed the concept and framework of artificial intelligence capability, designed its scale, and studied the impact of artificial intelligence capability on organizational creativity and organizational performance (Mikalef and Gupta, 2021). However, there are few studies on the specific impact of organizational AI capability on organizational creativity. First, AI capability is a new concept, which refers to the ability of a company to select, coordinate and utilize its AI-specific resources. However, existing research does not fully explain how AI capabilities contribute to the improvement of organizational creativity. For example, more current studies show that human-computer interaction researchers have built creativity support tools (CSTs) to enhance and expand human creativity, artificial intelligence researchers have developed computational creativity systems to test the cognitive theory of creativity (Davis, 2013), or through the completion of movie Morgan trailer, the enhancement of creativity by artificial intelligence system is reflected from the perspectives of coding and modeling (Smith et al., 2017). Second, existing research mainly elaborates on employee creativity from the personal level. As for employee creativity, studies have pointed out that employees based on emotional trust will not be afraid of being criticized for sharing their thoughts and ideas (Islam et al., 2021a). There are also studies that reveal the role of knowledge sharing in promoting organizations to establish creative culture (Islam and Asad, 2021). However, from the perspective of an organization, whether employees are willing to share knowledge is the key for AI to play a role in an organization. Therefore, exploring the influence path of AI capability on organizational creativity from the perspective of knowledge sharing is the key to make up for the deficiency of existing research. Third, the existing research lacks more exploration on the effect of organization-level factors to promote organizational knowledge sharing, in order to make organization accept artificial intelligence. Knowledge sharing among employees is carried out in the organization, and knowledge sharing can play a key role in the process of transforming personally owned knowledge into public knowledge (Chaudhary et al., 2021). Artificial intelligence is one of the main cornerstones for developing and strengthening knowledge sharing (Alansari and Mohamed, 2020). Therefore, it is particularly important to explore the role of more organizational factors in AI capability and knowledge sharing.

In order to make up for the shortcomings of the above research, we have done the following unique work: Firstly, we introduce the concept of artificial intelligence capability and examine its relationship to organizational creativity. Secondly, we explore the influence path between AI capability and organizational creativity from the perspective of knowledge sharing. The improvement of organizational creativity from the perspective of knowledge is the key to the embedding of AI. Finally, we think that the knowledge sharing as one of the critical paths, need to be taken seriously in the organization, so we explore the effect of group cohesion, further explore whether it helps to promote AI capability for knowledge sharing, this has important implications for complementing the combination of artificial intelligence and organizational behavior.

This paper has the following contributions: At the theoretical level, it promotes the understanding of the AI capability and explores the improvement path of organizational creativity. In addition, we emphasize the new influencing factor that promotes the improvement of organizational knowledge sharing under the embedding of AI, which refers to organizational cohesion. This is consistent with the previous thought that managers can better understand team cohesion, so as to help managers play the best in the team (Tekleab et al., 2016). At the practical level, this paper will help organizations to cultivate AI capability, improve organizational creativity from the perspective of knowledge sharing, and make organizations pay more attention to the role of organizational cohesion.

## Research Framework and Hypothesis Development

**FIGURE 1:**
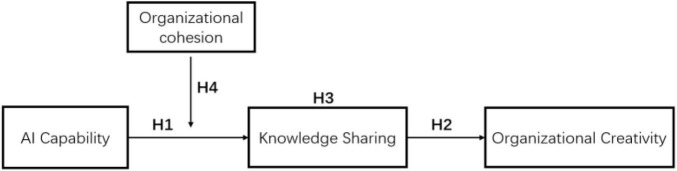
Artificial intelligence capability model.

This paper mainly carries on four levels of research: firstly, studies the relationship between AI capability and knowledge sharing; Secondly, the relationship between knowledge sharing and organizational creativity is studied. Thirdly, the mediating role of knowledge sharing between AI capability and organizational creativity is studied. Fourthly, the positive moderating effect of organizational cohesion on AI capability and knowledge sharing is studied. Based on the above theories, the theoretical model and hypothesis of this study are shown in Figure 1.

With the rapid development of science and technology, artificial intelligence technology has been infiltrated in all walks of life. Therefore, the concept of AI capability is put forward, which refers to the ability of a firm to select, orchestrate, and leverage its AI-specific resources. Scholars put forward the concept by referring to IT capability, resource-based theory that provides explanations for sustainable competitive advantage (Yu et al., 2020), and artificial intelligence research in the context of organizations. Artificial intelligence capability includes three main types of organizational resources: tangible resources, intangible resources and human resources. On this basis, a scale is formed to measure the relationship between AI capability and organizational creativity and performance in firms indicating that artificial intelligence capability can help improve organizational creativity and performance. Similarly, studies focusing on the development of artificial intelligence capabilities of public organizations further point out that the cultivation of artificial intelligence capabilities of public organizations needs to proceed from the organizational level rather than the individual level (Mikalef et al., 2021), making an important contribution to the research on artificial intelligence capabilities.

For an organization, knowledge plays a very important role in its development, and knowledge management (KM) is a basic process that can promote its development from the perspective of long-term sustainable development (Bhatt, 2000; Spender and Grant, 2015). Knowledge sharing in organizations, if properly managed, can enable organizations to gain and maintain long-term competitive advantages (Bhatt, 2000). As part of knowledge management, the concept of knowledge sharing (Pandey et al., 2021) is defined by scholars as “the process of exchanging task information, expert knowledge and feedback about procedures or products to create new knowledge or ideas, deal with problems and achieve common goals” (Kim and Park, 2017). Some scholars have found that good communication, open atmosphere and cooperation consciousness among people can create a working atmosphere that promotes knowledge sharing (Mooradian et al., 2006; Hooff et al., 2012; Nakano et al., 2013). Some scholars have found that knowledge sharing can promote the creativity of an organization (Lin, 2017), and other studies have shown that the support of management and supervisors is an important factor for the success of knowledge management and knowledge sharing plans (Teo, 2005).

Recent research on artificial intelligence and business value argues that organizations must cultivate a culture of teamwork, collective goals and shared resources in order to give full play to the value of AI technologies (Bughin et al., 2017). Strong inter-departmental coordination can promote the development of AI capability. Strong AI capability also means sustained relationship between departments and strong knowledge sharing culture of the organization. Based on the above literature, we make the following assumptions:

H1: Artificial intelligence capability has a positive impact on knowledge sharing.

Knowledge management refers to all the processes of organizational knowledge from creation to sharing, from transfer to transformation, and from explicit to implicit integration (Pandey et al., 2021). Knowledge sharing can also positively explain the positive impact of entrepreneurial leadership on employee creativity (Gao et al., 2021; Islam and Asad, 2021). Through knowledge management, organizations can improve organizational performance (Wang and Wang, 2012; Rasula et al., 2013). Knowledge sharers can experience the improvement of creativity through knowledge sharing, and knowledge sharing in the organization will have a positive impact on the organization. This can be attributed to the fact that knowledge sharing not only brings benefits to the knowledge sharers, but also promotes the recipients to develop more in-depth and extensive knowledge. Thus leading to better performance (Chiu et al., 2017). Through knowledge sharing, organizations can make better use of resources to innovate (Mahdi et al., 2018). Therefore, this paper makes assumptions:

H2: Knowledge sharing has a positive impact on organizational creativity.

AI capability means the extent to which a firm can make good use of and master its AI-specific resources (Mikalef and Gupta, 2021). Research shows that companies can realize benefits in organizational creativity by cultivating AI capabilities. But did not point out the specific influence path between the two. Therefore, this paper introduces knowledge sharing to explore its role in the relationship between artificial intelligence capability and organizational creativity.

AI capabilities enable organizations to invest in flexible data storage, technologies that process data quickly and run complex algorithms, and technologies that facilitate knowledge sharing (Pandey et al., 2021). In addition, higher AI capabilities are also reflected in an organization’s culture of good teamwork, consistent collective goals and shared resources, which has the potential to drive more active knowledge sharing, this is because the factors that influence members to hide or share knowledge depend on their personal intentions and organizational climate with knowledge explorers (Pereira and Mohiya, 2021). At the same time, for the learning of artificial intelligence technology, the community can improve the participation of citizens by using appropriate knowledge sharing tools (Alansari and Mohamed, 2020). Studies have shown that students in Pakistan can enhance their creativity by sharing knowledge with the required technological resources (Arif et al., 2022). Therefore, this paper makes assumptions:

H3: Knowledge sharing mediates the relationship between AI capability and organizational creativity.

It has been pointed out in the literature that AI contributes to the innovation process, and may even contribute to the knowledge base and innovation results of firms, while social cohesion around relationships will affect individuals’ willingness and motivation to share knowledge with others (Mcevily, 2003). In today’s social context, team cooperation and organizational collaboration are increasingly valued by people (Mathieu et al., 2017). As part of “cross-departmental coordination” in AI capabilities, collaboration between teams is more important. Some scholars have pointed out that members’ social interaction is positively correlated with the amount of knowledge shared by people, and the sense of social solidarity and group solidarity may significantly stimulate members’ willingness to share knowledge (Chiu et al., 2007). Research results show that group cohesion can effectively relieve the work pressure of nursing residents and improve their work quality. According to the social information processing theory, lack of team cohesion will affect the relationship between members of the organization by affecting the organizational atmosphere, which will lead to turnover intention (Raes et al., 2021). The strong relationship among team members contributes to the establishment of social networks and the formation of trust among people, resulting in effective knowledge sharing patterns (Wojciechowska-Dzięcielak, 2020). In addition, studies have shown that employees based on emotional trust feel safe and have more positive thoughts about knowledge sharing (Islam et al., 2021b). Therefore, based on the literature mentioned above, we propose the following hypotheses:

H4: Organizational cohesion has a positive moderating effect on the relationship between AI capability and knowledge sharing.

## Data and Research Methodology

### Scale Design and Data Sources

To test our hypothesis, this research adopts a questionnaire survey. At present, more and more firms have mastered artificial intelligence technology. Artificial intelligence technology has been widely used in various organizations, people from all walks of life can access to artificial intelligence technology. This paper mainly selects the Chinese high-tech firms as the survey object.

First, we get the information of high-tech firms from several large innovation ecosystems in China (Beijing Zhongguancun, Sino German Software Park, Suzhong Industrial Park, etc.), including name, address and contact persons. After that, we use e-mail, media software and other means to contact the firm and invite employees to fill in the questionnaires. We will state that the questionnaire is only for scientific research and there is no potential conflict of interest. After obtaining the consent of firms, we will officially send the network link address of the questionnaire. The data collection process lasted for 4 months, and 204 questionnaires were collected. However, in order to ensure the high quality and validity of the questionnaire, the invalid questionnaires need to be removed. The reasons include too short time of filling in the questionnaire (less than 5 min), empty and invalid information. Finally, a total of 189 high-quality questionnaires were obtained. The descriptive statistics of the sample are shown in Table 1.

**TABLE 1:** Results of descriptive statistics of the sample (*N* = 189).

Content	Category	Sample size	Proportion (%)
Gender	Male	108	57.14
	Female	81	42.86
Education Level	Senior high school (technical secondary) and below	79	41.80
	College or higher vocational college	50	26.46
	Bachelor	49	25.93
	Master and above	11	5.82
Firm nature	Private or private holding firms	114	60.32
	The foreign capital firm	22	11.64
	Wholly state-owned and holding firms	53	28.04
The firm scale	< 10	17	8.99
	10–100	89	47.09
	100–300	53	28.04
	> 300	30	15.87

### Variable Measurement

In order to ensure the reliability and validity of the measurement scale, mature measurement scale projects were selected in this study, and appropriate adjustments were made to the research scenarios.

Referring to the relevant researches of scholars, artificial intelligence capability consists of 38 questions (Mikalef and Gupta, 2021), and knowledge sharing consists of 4 questions (Rasula et al., 2012; Wang et al., 2016). Organizational creativity includes 5 question items (Mikalef and Gupta, 2021), and organizational cohesion (Henry et al., 1999) includes 7 question items.

The items in question in this paper were all scored on a 5-point Likert scale, that is, from 1 to 5 indicating never to complete agreement.

### Reliability and Validity Tests

Descriptive statistics and correlation coefficients are shown in Table 2. The results show that AI capability is positively correlated with knowledge sharing (*r* = 0.715, *p* < 0.01), knowledge sharing was positively correlated with organizational creativity (*r* = 0.473, *p* < 0.01), this result preliminarily supports hypothesis 1 and hypothesis 2.

**TABLE 2:** Descriptive statistics results with correlation coefficients.

Variables	Average	Standard deviation	1	2	3	4
1 AI capability	3.938	0.743	1			
2 Knowledge sharing	4.200	0.758	0.715**	1		
3 Organizational creativity	3.652	1.169	0.543**	0.473**	1	
4 Organizational cohesion	4.041	0.952	0.465**	0.430**	0.811**	1

* p < 0.05 ** p < 0.01.

As shown in Table 3, Cronbach α of the four variables are > 0.8, indicating that the questionnaire of this study has considerable reliability.

**TABLE 3:** Reliability test results.

Variables	Items	Cronbach α
AI capability	38	0.974
Knowledge sharing	4	0.867
Organizational creativity	5	0.952
Organizational cohesion	7	0.948

SPSSAU was used to directly test its validity through confirmatory factor analysis of the maturity scale (see Table 4). The results show that the standard load factors are all > 0.4, indicating that there is a strong correlation between potential variables and analysis item measures. The results showed that AVE values of the four factors are > 0.5, and CR values are > 0.7, showing good aggregation validity.

**TABLE 4:** Scale items and validity tests.

Factor (Latent variable)	Measurement items (significant variables)	Standard load factor
AI Capability (CR = 0.975, AVE = 0.520)	1. Our managers are able to understand business problems and to direct AI initiatives to solve them	0.494
	2. The AI project is given enough time for completion	0.505
	3. We have explored or adopted cloud-based services for processing data and performing AI and machine learning	0.558
	4. We have the necessary processing power to support AI applications (e.g., CPUs, GPUs)	0.585
	5. We have invested in networking infrastructure (e.g., firm networks) that supports efficiency and scale of applications (scalability, high bandwidth, and low-latency)	0.607
	6. We have explored or adopted parallel computing approaches for AI data processing	0.586
	7. We have invested in advanced cloud services to allow complex AI abilities on simple API calls (e.g., Microsoft Cognitive Services, Google Cloud Vision)	0.600
	8. We have invested in scalable data storage infrastructures	0.602
	9. Collaboration	0.747
	10. Collective goals	0.750
	11. Teamwork	0.793
	12. Same vision	0.795
	13. Mutual understanding	0.784
	14. Shared information	0.823
	15. Shared resources	0.819
	16. We are able to anticipate and plan for the organizational resistance to change	0.832
	17. We consider politics of the business reengineering efforts	0.839
	18. We recognize the need for managing change	0.841
	19. We are capable of communicating the reasons for change to the members of our organization	0.856
	20. We are able to make the necessary changes in human resource policies for process re-engineering	0.869
	21. Senior management commits to new values	0.879
	22. Our managers have a good sense of where to apply AI	0.556
	23. In our organization we have a strong proclivity for high risk projects (with chances of very high returns)	0.791
	24. In our organization we take bold and wide-ranging acts to achieve firm objectives	0.820
	25. We typically adopt a bold aggressive posture in order to maximize the probability of exploiting potential opportunities	0.812
	26. We have access to very large, unstructured, or fast-moving data for analysis	0.813
	27. We integrate data from multiple internal sources into a data warehouse or mart for easy access	0.821
	28. We integrate external data with internal to facilitate high-value analysis of our business environment	0.837
	29. We have the capacity to share our data across business units and organizational boundaries	0.643
	30. We are able to prepare and cleanse AI data efficiently and assess data for errors	0.569
	31. We are able to obtain data at the right level of granularity to produce meaningful insights	0.636
	32. The executive manager of our AI function has strong leadership skills	0.508
	33. Our managers are able to anticipate future business needs of functional managers, suppliers and customers and proactively design AI solutions to support these needs	0.540
	34. Our managers are capable of coordinating AI-related activities in ways that support the organization, suppliers and customers	0.586
	35. We have strong leadership to support AI initiatives and managers demonstrate ownership of and commitment to AI projects	0.606
	36. The AI initiatives are adequately funded	0.565
	37. The AI project has enough team members to get the work done	0.568
	38. Our managers are able to work with data scientists, other employees and customers to determine opportunities that AI might bring to our organization	0.546
Knowledge Sharing (CR = 0.869, AVE = 0.625)	1. Our employees exchange knowledge with their co-workers	0.802
	2. In their work, our employees rely on experience, skills, and knowledge	0.748
	3. In the relationship, we frequently adjust our shared understanding of end-user needs, preferences, and behaviors	0.827
	4. Our companies exchange information related to changes in the technology of the focal products	0.785
Organizational Creativity (CR = 0.956, AVE = 0.817)	1. Our organization has produced many novel and useful ideas (services/products)	0.919
	2. Our organization fosters an environment that is conductive to our own ability to produce novel and useful ideas (services/products)	0.923
	3. Our organization spends much time for producing novel and useful ideas (services/products)	0.912
	4. Our organization considers producing novel and useful ideas (services/products) as important activities	0.886
	5. Our organization actively produces novel and useful ideas (services/products)	0.851
Organizational Cohesion (CR = 0.949, AVE = 0.731)	1. This organization accomplishes things that no single member could achieve	0.754
	2. All members need to contribute to achieve the organization’s goals	0.808
	3. I think of this organization as part of who I am	0.815
	4. Members of this organization like one another	0.832
	5. I see myself as quite similar to other members of the organization	0.875
	6. In this organization, members rely on one another	0.912
	7. I enjoy interacting with the members of this organization	0.929

Kaiser-Meyer-Olkin (KMO) and Bartlett test results are shown in Table 5. KMO value is > 0.9, p value is > 0.01, indicating that the validity of the research data is feasible.

**TABLE 5:** KMO and Bartlett’ s test.

KMO value		0.935
Bartlett Sphericity test	Approximate cardinality	11385.757
	df	1431
	p-value	0.000

## Empirical Testing and Analysis

### Selection of Research Method

This study aims to explore the impact of AI capability on organizational creativity. Multi-level regression analysis is used to analyze the correlation between variables and their moderating effects. The core of multiple regression analysis is regression analysis, the difference is that hierarchical regression can be divided into multiple layers, with each layer adding more polynomials above the previous one. This method can solve the problem of whether more polynomials have explanatory power to the model. In addition, bootstrap sampling was used to test the mediating effect. The basic idea of bootstrap sampling is to construct the estimated confidence interval through multiple sampling and partial sample release when the complete sample is unknown. This method has high testing efficiency and does not impose restrictions on the intermediate sampling distribution.

### Correlation Test

In this paper, SPSSAU software is used to perform multi-level regression analysis on the hypothesis.

As shown in Table 6, two models are involved in this hierarchical regression analysis. The independent variables in Model 1 are control variables (gender, education level, firm nature, and firm scale), and AI capability is added to model 2 based on Model 1.

**TABLE 6:** Multilevel regression test results (Explanatory variable: Knowledge sharing).

Category	Variable	Model 1	Model 2
Control variables	Gender	−0.084 (−0.741)	−0.042 (−0.516)
	Education	−0.075 (−1.225)	−0.030 (−0.692)
	Firm nature	−0.071 (−1.090)	0.060 (1.271)
	Firm Scale	−0.021 (−0.312)	−0.022 (−0.458)
Independent variables	AI capability		0.735** (13.401)
Model explanatory degree	Sample size	189	189
	R^2^	0.028	0.509
	AdjustedR^2^	0.007	0.496
	F Value	*F*(4,184) = 1.333, *p* = 0.259	*F*(5,183) = 38.0176, *p* = 0.000
	ΔR^2^	0.028	0.481
	ΔF Value	*F*(4,184) = 1.333, *p* = 0.259	*F*(1,183) = 179.576, *p* = 0.000

*p < 0.05 **p < 0.01. The t-values are in parentheses.

The explanatory variable of this study is knowledge sharing. Model 1 tested the effect of control variables, and the model’s F-test showed that the model did not pass the F-test (*F* = 1.333, *p* > 0.05). This indicates that the four control variables of gender, education level, firm nature and firm scale have no significant influence on the logical path of knowledge sharing.

The results of model 2 show that after artificial intelligence capability is added to model 1, f value changes significantly (*p* < 0.05). This means that AI capabilities add explanatory meaning to the model. In addition, the R^2^ value increased from 0.028 to 0.509, suggesting that AI capabilities could explain knowledge sharing with a 48.1% power. Among them, the regression coefficient value of artificial intelligence capability is 0.735, significant (*t* = 13.401, *p* = 0.000 < 0.01), indicating that there is a significant positive correlation between artificial intelligence capability and knowledge sharing, supporting hypothesis 1.

As shown in Table 7, two models are involved in this hierarchical regression analysis. The independent variables in Model 1 are control variables (gender, education level, firm nature and firm scale), and knowledge sharing is added in Model 2 on the basis of Model 1.

**TABLE 7:** Multilevel regression test results (Explanatory variable: Organizational creativity).

Category	Variable	Model 1	Model 2
Control variables	Gender	−0.114 (−0.644)	−0.052 (−0.334)
	Education	0.034 (0.352)	0.088 (1.044)
	Firm nature	−0.075 (−0.736)	−0.023 (−0.252)
	Firm Scale	−0.093 (−0.891)	−0.078 (−0.840)
Independent variables	AI capability		0.734** (7.210)
Model explanatory degree	Sample size	189	189
	R^2^	0.012	0.231
	AdjustedR^2^	−0.009	0.210
	F Value	*F*(4,184) = 0.558, *p* = 0.693	*F* (5,183) = 10.967, *p* = 0.000
	ΔR^2^	0.012	0.219
	ΔF Value	*F*(4,184) = 0.558, *p* = 0.693	*F* (1,183) = 51.982, *p* = 0.000

*p < 0.05 **p < 0.01. The t-values are in parentheses.

The explanatory variable of this study was organizational creativity. Model 1 tested the effect of the control variable, and the F-test of the model showed that the model did not pass the F-test (*F* = 0.558, *p* > 0.05). This indicates that the four control variables of gender, education level, firm nature and firm size have no significant impact on the logical path of organizational creativity.

The results of model 2 show that after knowledge sharing is added to model 1, f value changes significantly (*p* < 0.05). This means that knowledge sharing adds explanatory meaning to the model. Furthermore, R2 increased from 0.012 to 0.231, suggesting that knowledge sharing accounted for 21.9% of organizational creativity. Among them, the regression coefficient value of knowledge sharing is 0.734, significance (*t* = 7.210, *p* = 0.000 < 0.01), indicating that there is a significant positive correlation between knowledge sharing and organizational creativity, supporting hypothesis 2.

### Intermediation Effect Test

In this paper, non-parametric percentage bootstrap method with bias correction was used to test the mediating effect (as shown in Table 8), with a confidence level of 95%.

**TABLE 8:** Summary of intermediary role test results.

Items	Total effect	a	b	Intermediary effect (a*b)	95% BootCI	Direct effect (c’)	Effectiveness ratio	Test conclusion
AI capability-Knowledge sharing-Organizational creativity	0.861**	0.727**	0.273*	0.199	−0.021 ∼0.270	0.663** (0.394 ∼ 0.931)	23.067%	Partial mediation

* p < 0.05 ** p < 0.01.

From AI capability to the process of organizational creativity, AI capability for the regression coefficient of knowledge sharing(a) and knowledge sharing for the regression coefficient of the organizational creativity(b) were significantly, and the direct effect(c’) is significant, a * b and c’ prime are both positive signs.

**FIGURE 2:**
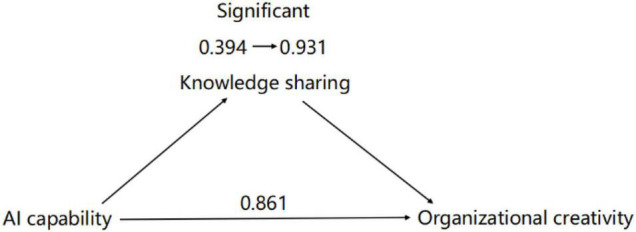
Intermediary role test results.

These results suggest that knowledge sharing plays a partially mediating role in the positive impact of AI capability on organizational creativity, H3 confirmed. The mediation role test results are shown in Figure 2.

### Adjustment Test

In this study, the influence of AI capability on knowledge sharing was studied to see whether the variable of organizational cohesion could play a moderating role, that is, whether there was a significant difference in the influence range of artificial intelligence capability on knowledge sharing when the variable of organizational cohesion was at different levels. And put four control variables into the model. Before the analysis, AI capability and organizational cohesion are first treated centrally, and dependent variable knowledge sharing and control variable are not treated.

Adjusted using multilevel regression analysis function test, this paper will be divided into three models, the model 1 variables including AI capability, model 2 on the basis of model 1 to join the organizational cohesion variables, model 3 on the basis of model 2 to join the interaction of the two items (the product term of AI capability and organizational cohesion).

As can be seen from Table 9, when the interference of organizational cohesion is not taken into account, the influence of AI capability on knowledge sharing is significant (*t* = 13.765, *p* = 0.000 < 0.05), indicating that artificial intelligence capability has a positive and significant influence on knowledge sharing. The interaction between AI capability and organizational cohesion was significant (*t* = −2.407, *p* = 0.017 < 0.05). This means that when AI capability affects knowledge sharing, the impact range of organizational cohesion is significantly different at different levels, indicating that organizational cohesion plays a positive moderating role in the impact of AI capability on knowledge sharing.

**TABLE 9:** Multilevel regression test results (Explanatory variable: Knowledge sharing).

Category	Variable	Model 1	Model 2	Model 3
Independent variables	AI capability	0.727** (13.765)	0.666** (11.281)	0.659** (11.277)
Mediating variables	Organizational cohesion		0.101* (2.215)	0.084(1.834)
	AI capability × Organizational Cohesion			−0.089* (−−2.407)
Model explanatory degree	Sample size	189	189	189
	R^2^	0.503	0.516	0.531
	AdjustedR^2^	0.501	0.511	0.523
	F Value	*F* (1,187) = 189.480, *p* = 0.000	*F* (2,186) = 99.172, *p* = 0.000	*F*(3,185) = 69.750, *p* = 0.000
	ΔR^2^	0.503	0.013	0.015
	ΔF Value	*F* (1,187) = 189.480, *p* = 0.000	*F* (1,186) = 4.906, *p* = 0.028	*F* (1,185) = 5.793, *p* = 0.017

*p < 0.05 **p < 0.01. The t-values are in parentheses.

## Discussion

Based on the concept of artificial intelligence capability, this paper develops a research model of artificial intelligence capability on organizational creativity, which is mediated by knowledge sharing and moderated by organizational cohesion. This model deepens the research on AI capabilities, and improves the research on artificial intelligence capabilities from the perspectives of knowledge sharing and organizational cohesion. According to the data, this model can well explain the influence of AI capability on organizational creativity under the mediation of knowledge sharing. The research results can be summarized into the following four aspects.

First of all, this paper introduces the concept of AI capability, explores the impact of AI capability on organizational creativity, and finds that AI capability contributes to the improvement of creativity. Artificial intelligence is hailed by many scholars and practitioners as a revolutionary and game-changing set of technologies in the business world (Ågerfalk, 2020). Some scholars have pointed out that AI can improve various key performance indicators at the organizational level. For example, other applications of AI are believed to improve the speed of data processing, thus reducing bottlenecks and improving overall operational efficiency (Ivanov and Webster, 2017). Through empirical research, this paper proves that there is a significant positive correlation between AI capability and organizational creativity. Strong artificial intelligence capability usually means that there is a strong culture of knowledge sharing within an organization, which can improve the degree of creation of new products. This conclusion plays an important role in promoting organizational creativity from the perspective of artificial intelligence, and also promotes the framework and research background of organizational behavior from the perspective of artificial intelligence. It can promote the understanding of the AI capability and make firms understand that artificial intelligence can promote the upgrading and creation of new products.

Second, this study explores the relationship between knowledge sharing and organizational creativity at the organizational level. Most of the existing researches study knowledge sharing and organizational creativity from the individual level (Perotti et al., 2021), but lack of relevant researches from the organizational level. Based on artificial intelligence capability, this paper examines the impact of organization-level knowledge sharing on organizational creativity, and confirms that there is a significant positive correlation between organization-level knowledge sharing and organizational creativity. This indicates that organizations with strong knowledge sharing culture contribute to the generation of organizational creativity.

Third, this paper explores the mediating role of knowledge sharing between AI capability and organizational creativity. Knowledge sharing within an organization can enable people to share and accept knowledge flow to improve individual and team performance, thus benefiting the entire organization (Bock et al., 2005; Cabrera and Cabrera, 2005; Jafari Navimipour and Charband, 2016). Some scholars have pointed out that knowledge sharing can help and cooperate with others to develop new ideas, solve problems and implement policies or procedures (Cummings, 2004; Sheng and Noe, 2010). Therefore, from the theoretical level, knowledge sharing practice can promote organizations to utilize knowledge-based resources in the creation of organizational capabilities such as knowledge application and innovation (Cabrera and Cabrera, 2005). Organizations with strong artificial intelligence capabilities can improve the efficiency of knowledge utilization by increasing the degree of knowledge sharing, thus enhancing the creativity of organizations. Through this study, it can be concluded that higher organizational AI capability can promote the efficiency and effectiveness of organizational knowledge sharing, and then improve the creativity of the whole organization. We have explored the impact of organizational AI capability on organizational creativity from the perspective of knowledge sharing, which can help to make up for the shortcomings of existing research.

Finally, this paper confirms the positive moderating effect of organizational cohesion on AI capability and knowledge sharing. From the organizational level, higher organizational cohesion will promote the impact of AI capabilities on knowledge sharing, which is of great significance for promoting knowledge sharing from the organizational perspective. We have emphasized the new influencing factor that promotes the improvement of organizational knowledge sharing under the embedding of AI, which refers to organizational cohesion. This is consistent with the previous thought that managers can better understand team cohesion, so as to help managers play the best in the team (Tekleab et al., 2016).

To summarize, at the theoretical level, we establish an AI capability model, verifies the relationship between AI capability and knowledge sharing, and verifies the relationship between knowledge sharing and organizational creativity. In addition, the mediating role of knowledge sharing between AI capability and organizational creativity, and the moderating role of organizational cohesion between AI capability and knowledge sharing are also studied. It complements the influence of artificial intelligence capability research on organizational creativity, and expands the boundary and application space of organizational cohesion and knowledge sharing theory at the organizational level, thus providing reference for improving organizational creativity from the perspective of organizations.

At the practical level, we provide suggestions on how to cultivate the ability of artificial intelligence and how to avoid the loss in the development of artificial intelligence; At the same time, this paper also provides a path to promote organizational creativity, and advocates the organization’s attention to knowledge sharing. This study puts forward necessary enlightenment for firm managers to cultivate organizational atmosphere and knowledge evolution under the virtuous circle. we also provide valuable suggestions for the development of AI capability in order to improve product innovation.

## Data Availability Statement

The raw data supporting the conclusions of this article will be made available by the authors, without undue reservation.

## Ethics Statement

The studies involving human participants were reviewed and approved by Jilin University. The patients/participants provided their written informed consent to participate in this study. Written informed consent was obtained from the individual(s) for the publication of any potentially identifiable images or data included in this article.

## Author Contributions

NL: designing. YaY and YuY: writing. AG: providing revised advice. All authors contributed to the article and approved the submitted version.

## Conflict of Interest

The authors declare that the research was conducted in the absence of any commercial or financial relationships that could be construed as a potential conflict of interest.

## Publisher’s Note

All claims expressed in this article are solely those of the authors and do not necessarily represent those of their affiliated organizations, or those of the publisher, the editors and the reviewers. Any product that may be evaluated in this article, or claim that may be made by its manufacturer, is not guaranteed or endorsed by the publisher.
